# A randomised controlled trial assessing the severity and duration of depressive symptoms associated with a clinically significant response to sertraline versus placebo, in people presenting to primary care with depression (PANDA trial): study protocol for a randomised controlled trial

**DOI:** 10.1186/s13063-017-2253-4

**Published:** 2017-10-24

**Authors:** George Salaminios, Larisa Duffy, Anthony Ades, Ricardo Araya, Katherine S. Button, Rachel Churchill, Tim Croudace, Catherine Derrick, Padraig Dixon, Christopher Dowrick, Simon Gilbody, William Hollingworth, Vivien Jones, Tony Kendrick, David Kessler, Daphne Kounali, Paul Lanham, Alice Malpass, Tim J. Peters, Derek Riozzie, Jude Robinson, Debbie Sharp, Laura Thomas, Nicky J. Welton, Nicola Wiles, Glyn Lewis

**Affiliations:** 1 Division of Psychiatry, University College London, 6th Floor Maple House, 149 Tottenham Court Road, London, W1T 7NF UK; 2 School of Social and Community Medicine, University of Bristol, Oakfield House, Oakfield Grove, Bristol, BS8 2BN UK; 3 Institute of Psychiatry, Psychology and Neuroscience, Kings’ College London, Denmark Hill, London, SE5 8AF UK; 4 Department of Psychology, University of Bath, 10 West, Bath, BA2 7AT UK; 5 Centre for Reviews and Dissemination, University of York, Heslington, York, YO10 5DD UK; 6 School of Nursing and Health Studies, University of Dundee, Airlie Place, Dundee, DD1 4HJ UK; 7 Institute of Psychology Health and Society, University of Liverpool, Waterhouse Building Block B, Liverpool, L69 3BX UK; 8 Department of Health Sciences, University of York, Seebohm Rowntree Building, Heslington, York, YO10 5DD UK; 9 Faculty of Medicine, University of Southampton, Aldermoor Health Centre, Southampton, SO16 5ST UK; 10 Department of Sociology, Social Policy and Criminology, University of Liverpool, Eleanor Rathbone Building, Bedford Street South, Liverpool, L69 7ZA UK

**Keywords:** Depression, Primary care, Antidepressants, Sertraline, Selective Serotonin reuptake inhibitors

## Abstract

**Background:**

Depressive symptoms are usually managed within primary care and antidepressant medication constitutes the first-line treatment. It remains unclear at present which people are more likely to benefit from antidepressant medication. This paper describes the protocol for a randomised controlled trial (PANDA) to investigate the severity and duration of depressive symptoms that are associated with a clinically significant response to sertraline compared to placebo, in people presenting to primary care with depression.

**Methods/design:**

PANDA is a randomised, double blind, placebo controlled trial in which participants are individually randomised to sertraline or placebo. Eligible participants are those who are between the ages of 18 to 74; have presented to primary care with depression or low mood during the past 2 years; have not received antidepressant or anti-anxiety medication in the 8 weeks prior to enrolment in the trial and there is clinical equipoise about the benefits of selective serotonin reuptake inhibitor (SSRI) medication. Participants who consent to participate in the trial are randomised to receive either sertraline or matching placebo, starting at 50 mg daily for 1 week, increasing to 100 mg daily for up to 11 weeks (with the option of increasing to 150 mg if required). Participants, general practitioners (GPs) and the research team will be blind to treatment allocation. The primary outcome will be depressive symptoms measured by the Patient Health Questionnaire-9 (PHQ-9) at 6 weeks post randomisation, measured as a continuous outcome. Secondary outcomes include depressive symptoms measured with the PHQ-9 at 2 and 12 weeks as a continuous outcome and at 2, 6 and 12 weeks as a binary outcome; follow-up scores on depressive symptoms measured with the Beck Depression Inventory-II, anxiety symptoms measured by the Generalized Anxiety Disorder-7 and quality of life measured with the Euroqol-5D-5L and Short Form-12; emotional processing task scores measured at baseline, 2 and 6 weeks; and costs associated with healthcare use, time off work and personal costs.

**Discussion:**

The PANDA trial uses a simple self-administered measure to establish the severity and duration of depressive symptoms associated with a clinically significant response to sertraline. The evidence from the trial will inform primary care prescribing practice by identifying which patients are more likely to benefit from antidepressants.

**Trial registration:**

Controlled Trials ISRCTN Registry, ISRCTN84544741. Registered on 20 March 2014. EudraCT Number: 2013-003440-22; Protocol Number: 13/0413 (version 6.1).

**Electronic supplementary material:**

The online version of this article (doi:10.1186/s13063-017-2253-4) contains supplementary material, which is available to authorized users.

## Background

Depression is a common condition that affects between 9% and 12% of the population at any one time [1] and is linked to higher rates of functional disability compared with most chronic medical illnesses [2]. Recent estimates suggest that depression is the leading cause of disability in high and middle-income countries [3, 4]. Depressive symptoms are usually managed within primary care and antidepressant medication is often the first line of treatment [5]. Overall, around 80% of people presenting with depression in UK general practice receive antidepressants [5, 6].

A dramatic rise of antidepressant medication prescription has been observed in recent years. There was an increase of 7.2% between 2013 and 2014 [7], and in 2014 over 57 million antidepressant prescriptions were issued in England, at a cost of £265 million [8]. Similar increases in antidepressant consumption have been observed in other high-income countries. [9]. Studies in UK primary care linked databases have found that the rate of new prescribing over this period remained stable and the increase in numbers of prescriptions arose because of an increase in the average duration of treatment with selective serotonin re-uptake inhibitors (SSRIs) [10, 11]. Given that individuals who receive antidepressant medication are likely to do so for long periods, there is a need to identify those individuals who are more likely to derive a clinical benefit from antidepressants.

One hypothesis to guide prescription is that the response to antidepressants (compared to placebo) is greater in those with more severe illness. Results from systematic reviews of aggregate data and from individual patient data have provided inconsistent support for this hypothesis [12–15]. One possible reason is that some studies had a narrow range of baseline severity. This will reduce the power to detect an interaction between baseline severity and response even in large databases. A different approach towards this question has been to restrict trials to people with “minor” depression not meeting the usual diagnostic criteria. A systematic review of studies of “minor” depression found no evidence for a beneficial treatment effect of antidepressants [16] consistent with the idea that there must be a lower threshold below which antidepressants are not effective. Given this conflicting evidence, we wish to test the hypothesis that the baseline severity of depression is likely to be a factor that can be used to predict benefit from antidepressant treatment.

The other possible factor that might be useful to predict response is the duration of depressive symptoms. Evidence suggests that antidepressants are effective in people with dysthymia [17] even though they do not meet the criteria for major depression. As a result the National Institute for Clinical Excellence (NICE) guidelines recommend SSRIs for “persistent subthreshold depressive symptoms” but give no definition of persistence [18].

Although general practitioners (GPs) have considerable expertise in identifying depression [19, 20], it is well-known that the measurement of depression is notoriously difficult using clinical assessment. This has led to the development of a whole range of standardised scales of varying length. Short, self-administered, questionnaires such as the PHQ-9 [21] are not sufficiently detailed to assess severity and duration accurately. On the other hand, semi-structured standardized interviews designed to assess depressive symptoms and diagnostic criteria are lengthy and often require the interviewer to use expert psychiatric judgments, a task that is not feasible in primary care settings [22]. If we are to provide guidance to GPs about the severity and duration of depression that may respond to treatment with antidepressants then we require a standardised assessment that is sufficiently detailed but could be used in primary care.

The final area we want to investigate is neuropsychological markers of antidepressant action. Harmer [23] and others have found consistently that antidepressants (both serotonin and noradrenaline drugs) acutely affect performance on emotion processing tasks, even though there is no subjective awareness of any change or improvement in mood. For example, memory of positive words is increased within a few days of taking antidepressants in healthy volunteers and in those with depression [24, 25]. The change in emotion-processing following antidepressant use is the reverse of that seen in depression and occurs before the onset of any clinical change in symptoms. Their theory suggests that the delay between the emotion-processing change and depressive symptoms depends upon the need to experience new events after the change of emotion processing has occurred. These markers of antidepressant response could be a factor that might be useful in predicting likely response to antidepressants.

Our overall aim is to improve the guidance for GPs and patients on who will benefit from treatment with antidepressants. We therefore propose to carry out a randomised controlled trial (RCT) to investigate the severity and duration of depressive symptoms that are associated with a clinically important response to sertraline in people presenting to primary care with depression. We plan to assess severity and duration of depression using a standardised measure (the Revised Clinical Interview Schedule (CISR) [22]) that can be self-administered on a computer and completed by the patient outside the consultation with the physician, so could potentially be used to guide assessment and prescription in primary care [26, 27]. Additionally, we want to investigate the effects of antidepressants on measures of emotion processing that might mediate the therapeutic effects and could also be a potential measure for predicting antidepressant response.

Our specific aims are:

1. To investigate the severity and duration of the depressive symptoms that are associated with a clinically important response to sertraline (compared to placebo) in people with depression
2. To investigate quality of life and the economic costs associated with response to treatment with sertraline
3. To test the hypothesis that sertraline will lead to an early change in emotion processing that will mediate any treatment effect on depressive symptoms

The long-term benefits of the trial will be in improving guidance/treatment recommendations for primary-care clinicians, thereby increasing the likelihood that a prescription will lead to clinical benefit, while reducing prescriptions that are not needed. We will include adult patients presenting in primary care with depressive symptoms/low mood, who are not currently on antidepressants (or in the previous 8 weeks) and the GP and/or patient are unsure whether there will be significant clinical benefit from taking SSRI antidepressants.

## Methods/design

### Study design

PANDA is a randomised, double-blind, placebo-controlled study in which eligible participants are individually randomised to sertraline or placebo. The sertraline will be encapsulated and matching placebo capsules will be produced in order to maintain the blind allocation during the study. Participants will be recruited from primary care practices across the UK in the areas surrounding our four trial sites: Bristol, London, Liverpool and York.

**Fig. 1:**
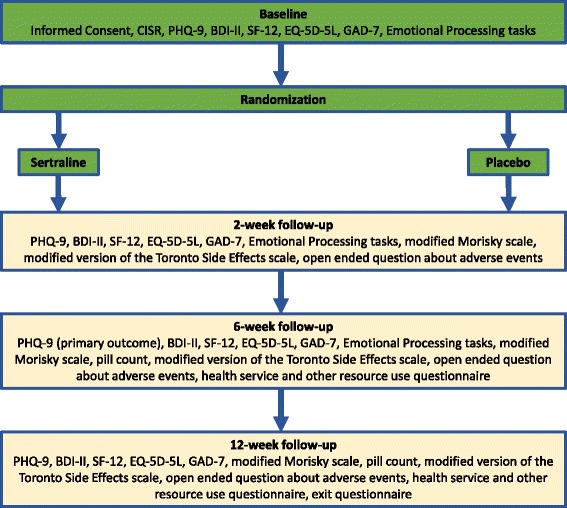
Summary of baseline and follow-up schedule for the PANDA trial. BDI-II Beck Depression Inventory-II; CISR Clinical Interview Schedule-Revised; EQ-5D-5L Euroqol 5D-5L; GAD-7 Generalized Anxiety Disorder-7; PHQ-9 Patient Health Questionnaire-9; SF-12 Short Form-12

Trial treatment will be for 12 weeks with research follow-up assessments at 2, 6 and 12 weeks (see Fig. 1 for a summary of the baseline and follow-up assessment schedule). The PHQ-9 [21] was selected as the primary outcome for depressive symptoms to avoid the observer bias associated with clinician-rated measures. The main treatment response of sertraline compared with placebo occurs within about 6 weeks, so in line with most antidepressant trials our primary outcome will be measured at the 6-week follow up. We also want to obtain an early assessment of adverse events, emotion processing and clinical response at 2 weeks as the first signs of improvement can occur at that point [28]. The 12-week assessment will provide evidence of any sustained benefit. In order to test our hypothesis about emotion processing and antidepressants [23], we will use two emotional processing tasks looking at (1) the recall of socially rewarding information and (2) reinforcement learning of reward and punishment (related to monetary rewards) that will be administered at baseline and then early on after 2 weeks, before we expect to see any clinical response.

Sertraline is an SSRI that is licensed for the treatment of depression and has a well-established efficacy profile. A recent network meta-analysis suggested that, if anything, it was more efficacious and better tolerated than most of the other SSRIs [29]. As a result it is one of the recommended SSRIs to use as a first choice in the treatment of depression [18] and is very widely prescribed in primary and secondary care in the UK and elsewhere in the world.

We want to keep the inclusion criteria pragmatic and broad to reflect the current dilemma in clinical practice, so uncertainty of GP and/or patient about the possible benefits of antidepressants is the key entry criterion for the trial. We will not impose any additional criteria of severity and duration ourselves.

### Inclusion and exclusion criteria

#### Inclusion criteria

People will be eligible for inclusion if they are between 18 and 74 years (inclusive) of age; have presented to primary care in the UK with depression at any point in the previous 2 years; and if there is clinical equipoise about the benefits of SSRI medication.

#### Exclusion criteria

People will be excluded if they have received antidepressant medication in the preceding 8 weeks; are unable to read, understand and/or complete questionnaires; suffer from other psychiatric disorders (i.e. psychosis, schizophrenia, bipolar disorder, mania, hypomania, dementia, eating disorder); suffer from major alcohol or substance abuse problems; are currently on contraindicated medication (i.e. monoamine oxidase inhibitors within the preceding 14 days or pimozide); suffer from poorly controlled epilepsy; have known allergies to sertraline, placebo or excipients; are concurrently enrolled in another investigational medicinal product (IMP) trial; are women who are currently pregnant or planning pregnancy or lactating; have severe hepatic impairment; suffer from bleeding disorders such as haemophilia, Christmas disease or von Willebrand’s disease, or have past medical history of bleeding gastric or duodenal ulcers or other significant bleeding disorders; or have had an episode of Torsade’s de Pointes.

### Recruitment of participants

Potential participants will be identified by GPs, who will invite patients at a consultation or perform a database search and mail out an invitation.

#### Method 1: in consultation

Given the patient’s permission for release of their contact details, the GP will directly refer potential participants by fax (or secure email) to the local trial centre. The researcher will contact the patient to confirm eligibility for the trial and arrange the baseline assessment visit.

#### Method 2: database search

GPs or practice administrative staff will carry out record searches to identify individuals for whom the GP has recorded low mood or depression symptoms during previous visits (within the last 2 years) and who are not currently on antidepressants. GPs will write to these individuals so they can consider joining the study.

The mail-out procedure will involve an initial letter sent by the GP surgery to the identified patients, inviting them to participate in the trial, followed by a reminder invitation letter if there is no response, and a further telephone call to patients who have not responded either to the initial or reminder invitations. Patients will only be contacted by individuals employed either directly by the GP practice or employed by National Health Service (NHS) organisations.

### Participant eligibility confirmation

Potential participants identified either at consultation or through a record search will receive a patient information sheet (PIS) that provides details of the study. If the patient agrees to be contacted by the research team, the GP will then complete and fax an eligibility form to the research team, confirming that the potential participant does not suffer from any psychiatric disorders or is on medication that would exclude them from the study. Potential participants will receive an additional phone call from a member of the research team to confirm eligibility for the trial. Provided that they do not meet any of the exclusion criteria, patients will be invited to a baseline assessment with a researcher either at their own home, the general practice or University premises.

### Baseline assessment

At the baseline meeting the researcher will explain the study in detail and obtain written informed consent to participate in the trial. Upon providing written consent, women of child-bearing age will carry out a pregnancy test. Participants will then undertake the following baseline assessments: a self-administered computerised clinical interview schedule (CISR) [22]; the PHQ-9 [21]; the Beck Depression Inventory-II (BDI-II) [30]; the Short Form-12 (SF-12) [31], the EQ-5D-5L [32]; the Generalized Anxiety Disorder-7 (GAD-7) [33]; and emotional-processing tasks [24, 34].

### Randomisation procedure and unblinding

Upon providing written consent and undertaking the baseline assessments, participants will be randomised to the trial by a member of the research team, and a letter will be sent to their GP to inform them of their patient’s enrolment.

Participants will be randomly assigned to one of the two treatments: (1) one × 50 mg encapsulated sertraline for 1 week followed by two × 50 mg encapsulated sertraline for up to 11 weeks and then for a 2-week tapering period or (2) identical placebo regimen. If participants have not responded to treatment after the 6-week follow-up assessment, the medication can be increased to three × 50 mg encapsulated sertraline or identical placebo in consultation with the Principal Investigator (PI).

Randomisation will be conducted by PRIMENT Clinical Trials Unit (CTU) using a remote computer-generated code (Sealed Envelope, https://sealedenvelope.com/). The randomisation will be stratified by severity and duration of depression and by research centre, with random block length. The pre-specified thresholds for stratification will be the CISR total severity score at baseline (0–11/12–19/20+) and depression duration (less than 2 years/2 years or more). The randomisation list will be held by Sealed Envelope. The random treatment allocation will then be sent to the central trial pharmacy (University Hospitals Bristol Pharmacy). Trial participants, care providers and all members of the research team will be blinded to the trial treatment allocation. Trial medication will be sent by the trial pharmacy to the participant’s GP (or participant’s home in exceptional circumstances) following the baseline and 6-week visit and at 10 weeks (for those on the 150 mg dose).

Upon receipt of study medication, participants will be provided with a contact card so that treating clinicians who may be external to the study team can be unblinded to treatment allocation in case of a clinical emergency. If unblinding is required, a formal request by a physician will be made to the trial pharmacy (through the 24 hour contact number provided on the contact card) that has a list of the participants’ treatment allocations. Study codes should only be broken for valid medical or safety reasons, for example in the case of a serious adverse event (SAE) where it is necessary for the responsible professional to know which treatment the patient is receiving before they can treat the patient. When possible, for treating professionals outside the research team, the unblinding request will be discussed with the investigating team (Chief Investigator (CI), local PI or delegate) so that a formal assessment can be undertaken. If in the opinion of the treating physician the code must be broken immediately, then this must be undertaken without further assessment. The treating physician will manage the medical emergency as appropriate upon receipt of the treatment allocation.

The CI/PI or delegate will record any breaking of the code and reasons for doing so on the case report form (CRF)/data collection tool and in the site file. Code breaks will also be documented in the final study reports. The CI/investigating team will notify the trial Sponsor (University College London (UCL)) on a yearly basis through the monitoring process. Where possible, members of the research team should remain unblinded.

When participants have ended the study and their outcome data have been entered into the database, they can request to be told their treatment allocation to placebo or active medication. This information will be provided to their GP by the central trial pharmacy, so the participant will need to consult their GP and any further treatment can be discussed during that consultation. The trial team will remain blind to this information.

### Treatment of participants

The IMP will be over-encapsulated sertraline and the matching placebo will be an identical capsule filled with an inert excipient. The placebo capsule will be identical to the encapsulated sertraline in dimensions and appearance, such that allocation concealment and blinding of the trial is maintained. Participants will be asked about adherence at all follow-up points and a pill count will be undertaken by a member of the research team at the 6-week and 12-week follow-up assessments. It will be requested that empty packaging and unused medicines are returned.

### Follow-up assessments

The research team will aim to conduct the follow-up assessments at 2, 6 and 12 weeks (see Table 1 for an overview of the study process). Participants will continue to be invited to follow-up assessments unless they have withdrawn from the trial. Research follow-up assessments will take place either at the participant’s home, the general practice or University premises. The date of the assessments will be recorded and the analysis plan will include measures to investigate the timing of the follow-up appointments. Participants will continue to be followed up even if they have stopped taking the study medication.

**Table 1:**
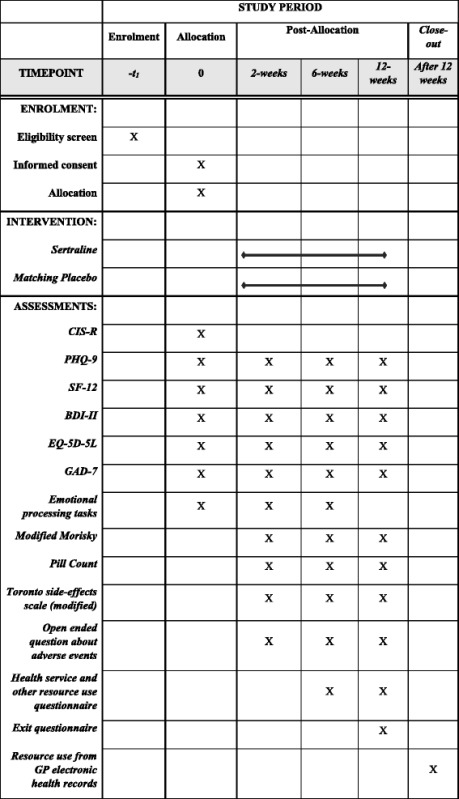
Full schedule of questionnaires – table showing the questionnaires used in the PANDA study

*BDI-II* Beck Depression Inventory-II, *CISR* Clinical Interview Schedule-Revised, *EQ-5D-5L* Euroqol 5D-5L, *GAD-7* Generalized Anxiety Disorder-7, *PHQ-9* Patient Health Questionnaire-9, *SF-12* Short Form-12, *GP* General Practitioner

The follow-up assessment schedule is as follows:

- At 2 weeks: the PHQ-9, BDI-II, SF-12, EQ-5D-5L, GAD-7, emotional processing tasks, modified Morisky adherence measure [35], side effects of antidepressant medication based on a modified version of the Toronto Side Effects scale as used in GENPOD [36]; open-ended question about adverse events and concomitant medication
- At 6 weeks: the PHQ-9, BDI-II, SF-12, EQ-5D-5L, GAD-7, emotional processing tasks, modified Morisky adherence measure and pill count, side effects of antidepressant based on a modified version of the Toronto Side Effects scale as used in GENPOD, open-ended question about adverse events and concomitant medication, health service and other resource use
- At 12 weeks: the PHQ-9, BDI-II, SF-12, EQ-5D-5L, GAD-7, modified Morisky adherence measure and pill count, side effects of antidepressant based on a modified version of the Toronto Side Effects scale as used in GENPOD, open-ended question about adverse events and concomitant medication, health service and other resource use
- After 12 weeks: primary healthcare use data (prescribed medication, primary care visits) extracted from GP electronic health records covering the full period of participation in the trial

### Data collection tools

#### CISR – revised clinical interview schedule

This is a self-administered, computerised assessment of psychiatric symptoms including depression, used at baseline only.

#### PHQ-9, BDI-II, SF-12, EQ-5D-5L and GAD-7

PHQ-9, BDI-II, SF-12, EQ-5D-5L and GAD-7 are self-administered written questionnaires.

#### Emotional processing tasks: memory for socially rewarding and socially critical information task [24]

This is assessed using a computerized task administered by a delegated member of the research team, either at the participant’s home, the primary care surgery or the university. At each time point, twenty likeable (e.g. cheerful, honest) and twenty dislikeable (e.g. untidy, hostile) personality characteristics are presented on a computer screen in a random order (each word is presented for 500 ms). Words are matched according to length, ratings of usage frequency and meaningfulness, and they differ at each time point. After each word, participants indicate whether they would “like” or “dislike” hearing someone describing them in this way (by pressing a key on the keyboard). At the end of the task, participants are asked to recall as many words as possible in 2 minutes. This is a surprise recall task (at baseline), to test incidental memory. The number of positive and negative words accurately recalled (hits) and the number of false responses (intrusions) are recorded.

#### Emotional processing tasks: reinforcement learning task [34]

This is assessed using a computerised task administered by a member of the research team. Each trial includes three events: the presentation of a fractal image, the presentation of a target and a probabilistic outcome. At the beginning of each trial, one of four possible fractal images is presented on a computer screen, which indicates whether the best choice in a subsequent target detection task is a go (pressing a key on the keyboard) or a no-go (withholding a response to the target). The fractal also indicates the valence of any outcome dependent on the participant’s behaviour (reward/no reward or punishment/no punishment). The meaning of fractal images (go to win, no-go to win, go to avoid punishment, no-go to avoid punishment) is randomised across participants, and participants have to learn these by trial and error. Participants are informed that the correct choice for each fractal image is either a go (button press) or no-go (withhold button press). Actions are required in response to a target circle that follows the fractal image. After a brief delay the outcome is presented (an upward arrow indicates a win, a downwards arrow indicates a loss and a horizontal bar indicates the absence of a win or a loss). On go-to-win trials, a button press is rewarded; on go-to-avoid-punishment trials, a button press avoids punishment; in no-go-to-win trials, withholding a button press is rewarded and in no-go-to-avoid-losing trials, withholding a button press avoids punishment. The task consists of 240 trials in total (60 trials per condition). The participant can win between £1 and £10. These data can be analysed using computational models [34].

### Outcome measures

#### Primary outcomes

1. Depressive symptoms measured using the PHQ-9 at 6 weeks as a continuous outcome

#### Secondary outcomes

1. Depressive symptoms with the PHQ-9 at 2 and 12 weeks
2. BDI-II as an alternative measure of depressive symptoms at all follow-up points
3. Anxiety symptoms measured using the GAD-7 at all follow-up points
4. Quality of life assessed using the EQ-5D-5L and SF-12
5. NHS costs associated with care, time off work and additional care and personal costs
6. Emotional processing tasks scores at baseline and 2 and 6 weeks

### Withdrawal from the trial

Participants may withdraw at any time but once dosing has occurred, every attempt should be made to continue assessments to ensure the safety of the individual concerned. Specific reasons for a participant withdrawing from the trial may be:

- Voluntary discontinuation by the individual, who is at any time free to discontinue his/her participation in the study, without prejudice to further treatment
- Risk to patients as judged by the investigator
- Severe non-compliance with the protocol as judged by the local site PI
- Incorrectly randomised individuals
- Adverse events

Any person who withdraws will always be asked about the reason(s) for withdrawal and the presence of any adverse events. If possible, they will be seen and assessed by the local site primary investigator or delegate. Adverse events will be followed up and trial discontinuation will be documented in the appropriate CRF pages. If possible, participants who discontinue the study medication before completion should undergo the assessments and procedures scheduled for the follow-up visits. Once participants have stopped their trial medication, they may not resume trial treatment.

A patient may withdraw from the follow-up visits or they may withdraw their consent for any data collected to be used. Patients will be encouraged to allow data that have been collected before withdrawal to be used in the analyses. However, if consent to use data is also withdrawn, then these data will be discarded. Patients withdrawing from the study will revert to the care of their GP.

### Trial medication

#### Packaging, labelling and dispensing

Medication packs will be labelled in accordance with applicable regulations and the Medicines and Healthcare products Regulatory Agency (MHRA) approvals. Each medication pack will have a medicine ID number, randomly generated to ensure sertraline and placebo medicine packs are indistinguishable (e.g. avoid all placebo packs being assigned an odd number) and thus maintain allocation concealment. This random number will be generated by the CTU and provided to the manufacturer who will use it as a unique identifier for the IMP packages and for the randomisation and code-break services.

The manufacturer will ship labelled and numbered packages to the pharmacy where the trial medication will be stored under controlled conditions. Storage will be secure, and there will be a delegation log for access, for which the pharmacy will take responsibility. The pharmacy will dispense individual patient packs and oversee the packaging and posting of those packs. After randomisation patient packs containing 6 weeks’ supply (90 capsules) of the trial medication will be posted by recorded delivery to the participant’s GP surgery or, in exceptional circumstances, their homes. After the 6-week assessment has been completed a further 6 weeks’ medication, including sufficient medication for the tapering period (90 capsules) will be posted by recorded delivery. All deliveries will be logged to ensure drug accountability. The trial medication will be shipped and stored in line with manufacturer’s stability data.

Full IMP accountability records will be maintained in the trial, and receipt, dispensing, distribution, return and destruction records will be maintained at the dispensing pharmacy. When the IMP arrives at the general practice it will be kept in a secure locked cabinet until collected by the participant. The receipt and collection of the IMP will be logged by the research team.

#### Concomitant medication

Sertraline should not be administered concomitantly with monoamine oxidase (MAO) inhibitors or within 2 weeks after discontinuation of MAO inhibitor therapy. Likewise at least 2 weeks should elapse before patients treated with sertraline are treated with MAO inhibitors. Participants in this study will not be treated with MAO inhibitors. Sertraline should not be administered concomitantly with pimozide.

Co-administration with other serotonergic active substances (L-tryptophan, triptans, tramadol, linezolid, lithium and St. John’s Wort – *Hypericum perforatum* – preparations) may lead to an incidence of serotonin-associated effects and participants will be advised not to take any of these medications for the duration of the trial.

Sertraline may increase the sedating properties of benzodiazepines and other sedatives (notably most antipsychotics, antihistamine H1 antagonists, opioids). Participants will be advised that caution should be exercised when these medicinal products are prescribed together with sertraline and they should be alert to the possibility of over-sedation.

Sertraline may increase the central nervous system (CNS) depressant effect of alcohol. Participants will therefore be advised to be cautious in their intake of alcohol while taking sertraline.

Co-administration of sertraline 200 mg daily with warfarin can result in a small increase in prothrombin time. If the participant is taking warfarin the GP will be asked to monitor prothrombin time when sertraline therapy is initiated or stopped and provide the results to the local PI.

The risk of bleeding may be increased when medicines acting on platelet function (e.g. nonsteroidal anti-inflammatory drugs (NSAIDs), acetylsalicylic acid and ticlopidine) or other medicines that might increase bleeding risk are concomitantly administered with SSRIs, including sertraline. The PI will decide whether the participant should be included in the study if they are also taking any NSAIDs or other medicines that might increase bleeding risk.

Participants will be allowed to take hypnotic medication along with the trial medication. The GP will confirm other medication that the participant is taking in order to assess contraindications to sertraline before the baseline assessment. Participants will be asked about any concomitant medication at all follow-up points.

#### Adverse events (AEs)

All AEs (untoward medical occurrences in a patient or clinical trial participant administered a medicinal product, which do not necessarily have a causal relationship with this treatment) will be recorded by a structured assessment in the follow-up assessments at 2, 6 and 12 weeks. If a participant consults the GP with a known AE it will be recorded in the medical notes only but not communicated to the PI unless specifically requested. This will include any AEs that occur during the tapering period after the 12-week assessment. As this trial is a trial of a licensed medication with a well-established safety profile that is used within its licensed indication, AEs will not be recorded in the CRF apart from those AEs of special interest included in the follow-up assessments.

Patients will be asked about serious adverse events (SAEs) at each visit using open-ended questions. All SAEs will be recorded in the CRF and the Sponsor’s SAE log. The SAE log will be reportable to the Sponsor once a year.

The PI or delegate at trial sites will inform the CI of any SAEs. The CI or an appropriate member of staff will complete the SAE form and will send it to the Sponsor via email within 24 hours of becoming aware of the event. The CI/PI may contact the patient’s GP, depending upon the nature of the SAE, to obtain more information on the adverse event. All suspected unexpected serious adverse reactions (SUSARs) must be notified to the Sponsor within 24 hours. The Sponsor will notify the main Research Ethics Committee (REC) and the MHRA of all SUSARs. SUSARs that are fatal or life-threatening must be notified to the MHRA and REC within 7 days after the Sponsor has learned of them. Other SUSARs must be reported to the REC and MHRA within 15 days after the Sponsor has learned of them.

### Statistical analysis

We will follow the Consolidated Standards of Reporting Trials (CONSORT) guidelines [37] in reporting and analysing our data. We will create a flow chart that will provide the number of potential participants who were screened, eligible, randomised and followed up at each time point.

Our primary outcome will be the PHQ-9 at 6 weeks. We will use a mixed-effects generalised linear modelling framework as it is appropriate for repeated measures data [38, 39]. The analyses will include adjustments for baseline PHQ-9 score and the stratification variables (severity in three categories, duration in two categories and centre). As we want to estimate the relationship between the CISR depression score and treatment, we will include this in the model (being aware of the possibility of collinearity) and will also use robust multivariate techniques.

We will carry out further analyses to assess the sensitivity of any results to the modelling assumptions and the covariance structure. This will be relevant to both the primary and secondary analyses. For the primary analysis we will carry out the analysis without the CISR depression severity measure in order to examine the robustness of the findings. We will examine the extent of individual heterogeneity in the longitudinal outcomes, if any. We will also examine interactions between baseline severity and treatment outcome within the model.

Other approaches to investigate include modelling the data with a linear regression model with different link functions in which log PHQ-9 is used for the outcome and baseline, and using a linear regression model with and without an interaction term. We will also carry out a sensitivity analysis by adjusting for any variables that are not balanced at baseline (themselves ascertained through descriptive statistics only). The duration of the depressive episode is measured before randomisation using the CISR assessment at baseline. The CISR includes a question about duration after each symptom section. After the depressive symptoms section the question asks about the following categories: less than 2 weeks, between 2 weeks and 6 months, between 6 months and 1 year, between 1 and 2 years, between 2 and 5 years, between 5 and 10 years and more than 10 years. Duration will be investigated as an additional parameter in the model used for the primary analysis.

Missing data: we will carry out sensitivity analyses to investigate the possible impact of missing data. The two main approaches will be to adjust for baseline variables associated with missing outcome data and also to use multiple imputation. We will make strenuous efforts to reduce the amount of missing data and in our power calculations have estimated that there will be up to 10% missing data at 6 weeks. In a similar previous trial (GENPOD) we obtained 91% follow up at 6 weeks [36].

Secondary analyses: the following secondary analyses will be conducted adjusting for the baseline measure of the outcome variable, stratification and minimisation variables:

- PHQ-9 score as a continuous outcome at 2, 6 and 12 weeks in a repeated measures analysis
- PHQ-9 score as a binary outcome where remission is defined as scoring < 10 on the PHQ-9 at 2, 6 and 12 weeks in a repeated measures analysis
- BDI-II score as a continuous outcome at 2, 6 and 12 weeks in a repeated measures analysis
- BDI-II score as a binary outcome (BDI-II <10) at 2, 6 and 12 weeks in a repeated measures analysis
- GAD-7 scores as a continuous outcome at 2, 6 and 12 weeks in a repeated measures analysis
- SF-12 physical and mental component scores at 2, 6 and 12 weeks in a repeated measures analysis
- Self-reported global improvement at 2, 6 and 12 weeks in a repeated measures analysis
- A mediation analysis to investigate whether scores on the emotional processing tasks at 2 weeks mediate a therapeutic effect on PHQ-9 at 6 and 12 weeks [40]

The choice of regression model will depend upon the outcome. Distributional assumptions appropriate for positive continuous outcomes will be investigated including those that model proportionate reduction of symptoms. The SF-12 is usually modelled using linear regression as this seems a good fit to the data. Logistic regression will be the used for the analysis of binary outcome data.

### Economic analysis

The economic evaluation will estimate costs from the NHS and social services perspective based on a review of medical notes and from responses to service and resource-use questionnaires. Unit costs will be obtained from published national sources where possible [41–43]. Cost will be expressed in pounds sterling, valued in the most recent available unit costs, adjusted for inflation where necessary. The EQ-5D-5L collected at baseline and 2, 6 and 12 weeks will be used to calculate quality adjusted life years (QALYs). EQ-5D-5L assesses quality of life in five domains and an index score is derived using a UK value set [44].

We will estimate the cost-effectiveness (cost per QALY) of sertraline at 12 weeks post randomisation. Differences between arms in costs and QALYs, and their confidence intervals, will be calculated using linear regression. We will also estimate (again with linear regression) the incremental net monetary benefit (INMB) [45] and associated confidence intervals, based on standard NICE cost-effectiveness thresholds [46]. We will estimate cost-effectiveness acceptability curves to depict the probability that sertraline is cost-effective at different values of this threshold. We will use net benefit regression [47] to explore the interaction between baseline symptom severity and duration and the cost-effectiveness of sertraline. If necessary, we will estimate cost-effectiveness models under multiple imputation of data, using imputation models as for the analysis of the primary trial outcome.

We will also conduct a descriptive, non-inferential “cost-consequences” analysis [48]. This will compare the primary and secondary outcomes of the trial, and the QALY outcomes described above, with costs to the NHS, social services and individual participants.

### Justification of sample size

We propose to use a model that will estimate a proportionate reduction in PHQ-9 score as the treatment effect, so we can analyse the data without using an interaction term. The results from previous meta-analyses suggest that the effect size of SSRIs versus placebo is about an 11% reduction in the Hamilton Rating Scale for Depression (HAM-D) score [49]. In the Fournier meta-analysis [12], the reduction is about 17% (personal communication, Jay Fournier). We have therefore taken the 11% estimate as the more conservative option in order to inform our power calculation. Our best estimate of the minimal clinically important difference (MCID) from the PANDA cohort study is that this corresponds to a 14 percentage points (95% CI 10 to 17 percentage points) reduction in score on the PHQ-9 (unpublished results but see [50] for a description of the approach). Therefore giving the power for effect sizes of 11 and 14 percentage points is reasonable and conservative in the light of the confidence limits and the previous results from the systematic review.

An 11 percentage-point difference corresponds to a difference in proportions of 0.89 and this is −0.117 on the natural logarithm scale. A 14% difference corresponds to 0.86 and −0.15 on the natural logarithm scale. We have estimated the SD of the logarithm of PHQ-9 scores using existing data from the PANDA cohort study in a Poisson regression in which the follow-up PHQ-9 scores are the outcome and the baseline scores are an offset variable. This led to an estimate of SD of 0.32 − 0.34 for the log PHQ9. Given the uncertainty in estimating SDs we have also included estimates assuming an SD of 0.4. We have assumed a normal distribution as this will be a good approximation to the Poisson distribution for the sample sizes involved.

Table 2 gives our estimates of sample size assuming a significance level of 5% (two sided) and power of 90%. Given the uncertainties surrounding many of the assumptions, we chose to recruit a sample of 547 participants.

**Table 2:** The sample size estimates for the PANDA trial with a variety of assumptions

Percentage point reduction	Natural log of reduction	SD estimate	Total sample size	Allowing for 10% attrition
11	−0.117	0.34	366	407
14	−0.150	0.34	216	240
11	−0.117	0.4	492	547
14	−0.150	0.4	300	333

### Data handling and quality assurance

The trial sponsor is UCL and it takes primary responsibility for ensuring that the design of the study meets appropriate standards and that arrangements are in place to ensure appropriate conduct and reporting. A monitoring plan has been agreed with the Sponsor. The trial will be run in accordance with Good Clinical Practice (GCP) and current regulatory guidance. All data at the site will be handled according to the Data Protection Act 1998 and UCL Information Security Policy and Trust Information Governance Policy. The investigators have full access to all the data and are under no restrictions in their use of the data. We are open to approaches from bona fide researchers to have access to the data providing this is consistent with our ethics and regulatory approvals.

### Publication policy

The funder, National Institute for Health Research (NIHR), is informed of the publications before they are submitted to journals. The co-applicants have agreed a publication policy. Publications will conform to the International Committee of International Journal Editors (ICMJE) guidelines for reporting and authorship.

### Ethics, regulatory approvals and reporting

The Sponsor will ensure that the trial protocol, patient information sheet, consent form, GP letter and submitted supporting documents have been approved by the appropriate regulatory body (MHRA in the UK) and the main research ethics committee, prior to any patient recruitment. The protocol and all agreed substantial protocol amendments were documented and submitted for ethical and regulatory approval prior to implementation. Ethical approval was obtained from the National Research Ethics Service committee, East of England - Cambridge South (ref: 13/EE/0418). Clinical trial authorization was given by the MHRA. The trial Sponsor is UCL. The trial has been registered with EudraCT Number 2013-003440-22, ISRCTN84544741. The protocol adheres to the Standard Protocol Items: Recommendations for Intervention Trials (SPIRIT). The SPIRIT Checklist is available as an Additional file 1.

It is the responsibility of the CI/PI or designee at each site to ensure that all subsequent amendments gain the necessary approval. This does not affect the individual clinician’s responsibility to take immediate action if thought necessary to protect the health and interest of individual patients.

The trial investigators and institutions will permit trial-related monitoring, audits, REC review, and regulatory inspections, providing direct access to source data/documents. Trial participants are informed of this during the informed consent discussion.

Within 90 days after the end of the trial, the CI/Sponsor will ensure that the main REC and the MHRA are notified that the trial has finished. If the trial is terminated prematurely, those reports will be made within 15 days after the end of the trial. The CI will supply the Sponsor with a summary report of the clinical trial, which will then be submitted to the MHRA and main REC within 1 year after the end of the trial.

There is a Trial Steering Committee chaired by Professor Carolyn Chew-Graham of Keele University. The other independent members are Professor Ian Anderson, Dr Evan Kontopantelis, Professor Anne Rogers and Mr Paul Lanham. The Independent Data Monitoring Committee is chaired by Prof Chris Williams of Glasgow University. The other members are Professor Richard Byng and Dr Obi Ukoumunne.

### Insurance

The UCL holds insurance against claims from participants for injury caused by their participation in the clinical trial. Participants may be able to claim compensation if they can prove that UCL has been negligent.

## Discussion

PANDA is a pragmatic primary-care trial with broad inclusion criteria, aiming to address important aspects of current clinical practice. Trial participants will be recruited from a wide range of primary care settings across the four study sites, based in urban, rural, affluent and deprived areas across the UK, thus minimizing selection bias. Given that the main treatment effect of sertraline occurs within 6 weeks, our primary outcome will be measured at the 6-week follow-up assessment. We have included a follow-up assessment at 2 weeks to obtain an early account of potential adverse events and the first signs of clinical response and at 12 weeks to obtain evidence of any sustained benefits of antidepressant treatment. Self-report measures will be used to assess clinical outcome in order to eliminate potential observer bias. As common adverse effects of sertraline may lead to inadvertent unblinding, we will ask trial participants at each follow-up period to indicate to which treatment arm they believe they have been allocated.

Given the increasing rates of antidepressant prescription across the UK and the clinical challenge associated with accurately assessing depressive symptoms during short consultation appointments, there is a need to identify which patients presenting to primary care with depression are more likely to benefit from a course of antidepressants. Our study will use a simple, self-administered, computerised assessment to establish the severity and duration of depressive symptoms and investigate any association with a clinically significant response to sertraline. The evidence from the trial will be used to inform primary-care prescribing practice by identifying which patients are more likely to benefit from antidepressants and using assessments that have the potential to be used in primary care.

## Trial status

The trial began recruiting participants in January 2015 and will be ongoing until August 2017. At the time of writing (June 2017), 161 general practices have been actively involved in PANDA and 573 participants have been randomised into the study. It is expected that data collection will be completed in November 2017.

## Additional file

Additional file 1: SPIRIT 2013 Checklist: recommended items to address in a clinical trial protocol and related documents. (DOC 122 kb)

## Abbreviations

AE: Adverse event
BDI-II: Beck Depression Inventory-II
CI: Chief Investigator
CISR: Clinical Interview Schedule-Revised
CNS: Central nervous system
CONSORT: Consolidated Standards of Reporting Trials
CRF: Case report form
EQ-5D-5L: Euroqol 5D-5L
GAD-7: Generalized Anxiety Disorder-7
GCP: Good Clinical Practice
GENPOD: Genetic and Clinical Predictors of Treatment Response in Depression study
GP: General Practice/Practitioner
HAM-D: The Hamilton Rating Scale for Depression
IMP: Investigational medicinal product
INMD: Incremental net monetary benefit
MAO: Monoamine oxidase inhibitor
MCID: Minimal clinically important difference
MHRA: Medicines and Healthcare Products Regulatory Agency
NICE: National Institute for Clinical Excellence
NIHR: National Institute for Health Research
NHS: National Health Service
NSAID: Nonsteroidal anti-inflammatory drug
PANDA: What Are the Indications for Prescribing Antidepressants that will Lead to a Clinical Benefit?
PHQ-9: Patient Health Questionnaire-9
PI: Principal Investigator
PIS: Patient information sheet
PRIMENT CTU: PRIMENT Clinical Trials Unit
QALY: Quality adjusted life year
REC: Research Ethics Committee
SAE: Serious adverse event
SD: Standard difference
SF-12: Short Form-12
SSRI: Selective serotonin re-uptake inhibitor
SUSAR: Suspected unexpected serious adverse reaction
UCL: University College London
